# A Case of Atypical Familial Mediterranean Fever With Pseudopolyposis-Like Mucosal Changes in the Ileum

**DOI:** 10.7759/cureus.57566

**Published:** 2024-04-03

**Authors:** Hiroyuki Ariga, Maaya Nakashima, Akiko Mikada, Etaro Hashimoto, Junya Kashimura

**Affiliations:** 1 Department of Gastroenterology, Mito Kyodo General Hospital, Mito, JPN; 2 Department of General Medicine, Mito Kyodo General Hospital, Mito, JPN; 3 Department of Family Medicine, Mito Kyodo General Hospital, Mito, JPN

**Keywords:** periodic fever, inflammatory bowel disease, colchicine, mefv gene, familial mediterranean fever

## Abstract

A 15-year-old male patient presented with recurrent fever. Three months prior, he experienced repeated fevers of 38°C, headaches, and malaise for three days. He experienced repeated fevers over 38°C for >72 hours two weeks prior to the current visit. A computed tomography scan showed enlarged lymph nodes around the ileum, suggesting familial Mediterranean fever (FMF) or inflammatory bowel disease. Endoscopic examination revealed a deformed Bauhin valve and inflammatory changes in the ileum, making inflammatory bowel disease unlikely. Thus, FMF was suspected, and after a thorough explanation, the patient was treated with colchicine, and his symptoms improved. Genetic testing revealed a mutation in the *MEFV* gene P369S-R408Q, and atypical FMF was diagnosed.

## Introduction

Familial Mediterranean fever (FMF) is an inherited auto-inflammatory disease that produces periodic fevers, serositis (pleurisy and peritonitis), and various skin lesions [1,2]. The differential diagnosis of periodic fever syndrome can be broadly classified as hereditary or non-hereditary [3]. FMF, cryopyrin-associated periodic syndrome, and high IgD syndrome (mevalonic acid kinase deficiency) are the most common types of hereditary periodic fever. Causes of non-hereditary periodic fever include unusual infections, malignancy, precancerous conditions, adult-onset Still’s disease, stomatitis with aphthae, pharyngitis, and cervical lymphadenitis. FMF is often manifested in childhood and is the most common adult-onset hereditary periodic fever syndrome, but there have been reports of cases complicated by gastrointestinal lesions resembling inflammatory bowel disease [4,5]. Symptoms include fever for one to three days, chest and back pain on inspiration, severe abdominal pain, and other types of serous inflammation. In the presence of symptoms, it is important to evaluate the response to colchicine [2,6].

## Case presentation

**Figure 1:**
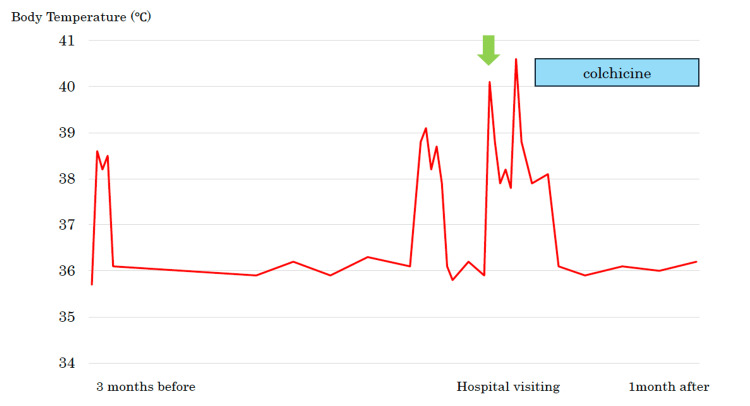
The patient had recurrent periodic fevers by the time he came to the hospital. His symptoms improved rapidly after colchicine administration.

A 15-year-old male patient presented at the clinic with the chief complaint of recurrent fever. Three months prior to the current visit, he had experienced fevers of 38°C, headaches, and malaise for three days. At that time, he visited a local doctor, who performed COVID-19 and influenza antigen tests that were both negative, and his fever resolved spontaneously. One month prior to the current visit, he had experienced a fever lasting five days and visited a local doctor. COVID-19 and influenza tests performed at that time were negative again. The fever had continued for 10 days prior to visiting our hospital, sometimes exceeding 40°C, and he was referred to our hospital for a thorough examination (Figure 1).

The patient complained of fatigue, and a fever was noted on physical examination. Other physical findings such as oral cavity, skin, joints, chest, and abdomen were normal, and there were no symptoms of abdominal pain, diarrhea, or bloody stools. Blood tests showed elevated inflammatory markers, CRP levels, and erythrocyte sedimentation rate (ESR). Serum amyloid A levels and hemoprecipitation were also elevated. Blood, stool, and urine culture tests were negative, and T-spot evaluation for tuberculosis was also negative (Table 1).

**Table 1:** Laboratory data. SAA: Serum amyloid A; ESR: erythrocyte sedimentation rate; GGT: gamma-glutamyl transpeptidase; TIBC: total iron-binding capacity; UIBC: unsaturated iron-binding capacity; T-Bil: total bilirubin; AMY: amylase test; ALB: serum albumin test; BUN: blood urea nitrogen

Laboratory Data							
WBC	5300/μL		Ca	9.3 mg/dL		TIBC	319 μg/dL
RBC	503×10⁴/μL		ALP	174 U/L		UIBC	300 μg/dL
Hb	12.6 g/dL		GGT	10 U/L		Fe	19 μg/dL
PLT	22.0×10⁴/μL		AST	26 U/L		Ferritin	318.7 ng/mL
			ALT	17 U/L			
TP	6.7 g/dL		T-Bil	0.6 mg/dL		SAA	214 μg/mL
ALB	4.1 g/dL		LDH	347 U/L			
BUN	10 mg/dL		AMY	124 IU/l		ESR	47 mm/hour
Cre	0.77 mg/dL		CRP	4.45 mg/dL		T-spot	Negative
UA	5.3 mg/dL						
Na	132 mEq/L		TSH	1.94 μIU/mL			
Cl	95 mEq/L		Free-T3	2.55 pg/mL			
K	3.8 mEq/L		Free-T4	1.55 ng/dL			

**Figure 2:**
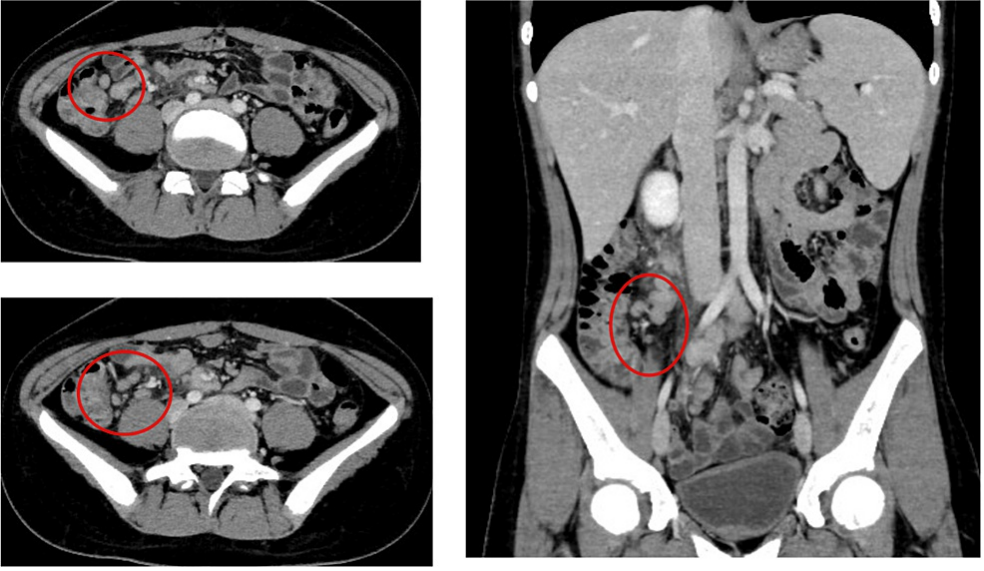
Contrast-enhanced computed tomography depicting enlarged lymph nodes around the ileum and ileocecal region (red circles).

Before visiting the hospital, he had black, watery stools, and contrast-enhanced computed tomography depicted enlarged lymph nodes around the ileum and ileocecal region (Figure 2).

**Figure 3:**
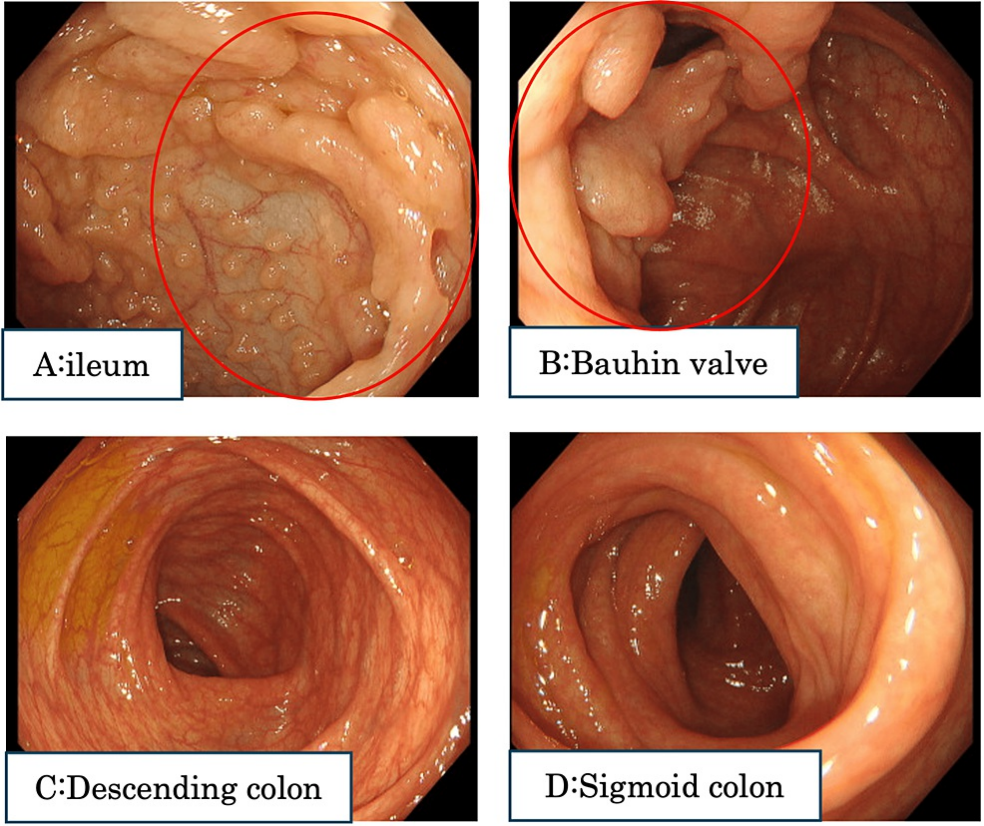
Lower gastrointestinal endoscopy revealed a deformed Bauhin valve and a finding that appeared to be pseudopolyposis. There were no obvious abnormalities in other parts of the colon.

Lower gastrointestinal endoscopy was performed to rule out inflammatory bowel disease. Endoscopy revealed a deformed Bauhin valve and pseudopolyposis-like changes in the ileum (Figure 3). There were no obvious abnormalities in other parts of the colon.

Histopathologic examination of a tissue biopsy taken at the time of endoscopy showed infiltration of inflammatory cells, no granuloma species, and a culture of intestinal fluid was negative for pathogenic organisms. Intestinal Behcet's disease was raised as a differential, but HLA-B51 and HLA-A26 were negative. Although he had no family history, FMF was suspected, and after a thorough explanation, colchicine was administered, and the patient's symptoms improved. Tel-Hashomer criteria also showed fever as a major criterion and rapid improvement with colchicine as a minor criterion, and genetic testing revealed a mutation (P369S-R408Q) in the *MEFV *gene. Based on the above, a diagnosis of atypical FMF was made.

## Discussion

FMF is a disease characterized by periodic fever, serositis, arthritis, and various skin lesions, and it is caused by mutations in the *MEFV* gene [2,7-10]. Clinical diagnosis of FMF has historically been based on the Tel-Hashomer criteria published in 1997 [11]. Alternative diagnostic criteria for FMF, revised from the Tel-Hashomer criteria, are valid in ethnic groups with a high incidence of FMF [11]. The original Tel-Hashomer criteria are valid in ethnic groups with a low incidence of FMF, such as Japan, where cases are commonly sporadic. In ethnic groups with a low incidence of FMF, atypical cases are more common. *MEFV* gene analysis is a useful diagnostic tool when FMF is suspected. *MEFV* gene exon 10 mutations are rare in atypical FMF, whereas mutations in exon 1 (E84K), exon 2 (El48Q, L110P-E148Q, R202Q, and G304R), exon 3 (P369S-R408Q), and exon 5 (S503C) are often present. Colchicine, the first-line medication as treatment for FMF, effectively improves FMF symptoms, even in atypical cases. In the current patient, FMF was suspected, colchicine was administered, and his symptoms rapidly improved. Concurrent genetic testing was performed, and atypical FMF was diagnosed.

Fever is a clinical symptom encountered in daily practice, but there are many potential causes, such as infections, malignancies, rheumatisms, and autoimmune diseases. We believe that pediatric diseases should be considered in the case of a 15‑year-old male. In a survey of children with fever of unknown cause in Japan, 11% of patients with a final diagnosis had periodic fever [12].

FMF is most frequently associated with high fever and abdominal pain due to peritonitis and rarely with intestinal lesions due to predominant serositis. Atypical FMF tends to involve few *MEFV* exon 10 mutations and many heterozygous mutations [4,5]. In patients with FMF and gastrointestinal lesions, typical cases account for approximately 30% and atypical cases account for approximately 70% [4]. The gastrointestinal sites affected by FMF often include the colon and jejunum, although the entire gastrointestinal tract may be affected. Almost all patients had abdominal symptoms such as abdominal pain, diarrhea, and bleeding, and arthritic symptoms were present in 34% of patients, but not in the present case. Characteristic findings of lower gastrointestinal lesions include ulcerative colitis-like mucosal abnormalities, present in more than 50% of cases. Rectal lesions are often absent, and longitudinal ulcerative lesions and stenoses similar to those seen in patients with Crohn's disease are often present, including in the ileum, as in this case [4,5]. Some patients with FMF have been reported to be undiagnosed and treated with biological agents for inflammatory bowel disease, with recurrent febrile peritonitis of unknown origin, peritonitis, pleurisy and pleuritis. It is important to consider FMF as a differential diagnosis in cases presenting with recurrent febrile peritonitis of unknown cause and pleuritic symptoms and carry out a thorough investigation.

## Conclusions

The patient presented with perigastrointestinal fever and *MEFV* genetic testing revealed a mutation in exon 3 (P369S-R408Q). Symptoms rapidly improved after administration of colchicine and a diagnosis of atypical FMF was made. Although there have been reports of cases of intestinal mucosal changes in FMF, as in the present case, the frequency and natural history of these changes remain unclear. We consider this a valuable case to be reported.
